# SARS-CoV-2 Survival in Common Non-Alcoholic and Alcoholic Beverages

**DOI:** 10.3390/foods11060802

**Published:** 2022-03-10

**Authors:** Mo Jia, Jonathan D. Joyce, Andrea S. Bertke

**Affiliations:** 1 Population Health Sciences, Virginia Maryland College of Veterinary Medicine, Virginia Polytechnic Institute & State University, Blacksburg, VA 24061, USA; moj@vt.edu; 2 Translational Biology, Medicine & Health, Virginia Polytechnic Institute & State University, Blacksburg, VA 24061, USA; jjoyce84@vt.edu; 3 Center for Emerging Zoonotic and Arthropod-Borne Pathogens, Virginia Polytechnic Institute & State University, Blacksburg, VA 24061, USA

**Keywords:** SARS-CoV-2, COVID-19, transmission, beverages, plaque assay

## Abstract

SARS-CoV-2, the causative agent of COVID-19, is known to be transmitted by respiratory droplets and aerosols. Since the virus is shed at high concentrations in respiratory secretions and saliva, SARS-CoV-2 would also be expected to be transmitted through activities that involve the transfer of saliva from one individual to another, such as kissing or sharing beverages. To assess the survival of infectious SARS-CoV-2 in common beverages, we quantified infectious virus by plaque assays one hour after inoculation into 18 non-alcoholic and 16 alcoholic beverages, plus saliva, and also 7 days later for 5 of these beverages. SARS-CoV-2 remains infectious with minimal reductions in several common beverages, including milk and beer. However, cocoa, coffee, tea, fruit juices, and wine contain antiviral compounds that inactivate SARS-CoV-2. Although hard liquors containing 40% alcohol immediately inactivate SARS-CoV-2, mixing with non-alcoholic beverages reduces the antiviral effects. In summary, SARS-CoV-2 can be recovered from commonly consumed beverages in a beverage type and time-dependent manner. Although aerosol or droplet transmission remains the most likely mode of transmission, our findings combined with others suggest that beverages contaminated with SARS-CoV-2 during handling, serving, or through sharing of drinks should be considered as a potential vehicle for virus transmission.

## 1. Introduction

SARS-CoV-2, the causative agent of COVID-19, is a highly transmissible virus responsible for more than 5.8 million deaths worldwide as of February 2022 [1]. One of the most important aspects to understand about a newly emerged pathogen is its mode of transmission, which allows for appropriate infection control and mitigation strategies to prevent, or at least reduce, the risk of transmission. Since SARS-CoV-2 will likely become an endemic virus throughout the world, a thorough understanding of all potential routes of transmission is needed to identify situations or venues that may promote a greater risk of on-going transmission or localized outbreaks that may spread beyond the community.

Social gatherings have been implicated as the source of multiple outbreaks of COVID-19, with attendees of these events carrying the virus back to their home locations or bringing the virus to a specific community. For example, a multistate outbreak of SARS-CoV-2 infections occurred after a large gathering in Provincetown, Massachusetts, in the United States [2]. Universities across the United States have also experienced a rise in cases due to congregate living, close interactions among people from various geographical regions, and social activities. Even among households, social gatherings such as birthday parties have produced higher rates of infections compared to households without a birthday [3].

SARS-CoV-2 is known to be transmitted person-to-person by aerosols or respiratory droplets consisting of saliva, nasal secretions, or sputum generated during coughing, sneezing, heavy breathing, singing, or talking [4,5,6,7]. A person may generate 1.23 × 10^5^ copies of SARS-CoV-2 in a single cough and saliva contains 10^4^ to 10^8^ copies of viral RNA per mL, with the highest viral loads prior to or just after symptom onset [7,8]. Although the virus concentration in secretions declines over time, viral RNA has been detected in saliva 25 days after symptom onset and viable virus has been isolated 15 days after onset, providing a broad range of time during which the virus could be transmitted to others [8,9,10]. Since SARS-CoV-2 is present in saliva, one would expect that in addition to droplets and aerosols, the virus would also be transmitted through activities that involve the transfer of saliva from one individual to another, such as kissing or sharing beverages.

Although SARS-CoV-2 is not considered to be a foodborne pathogen according to the World Health Organization (WHO), United States Food and Drug Administration (FDA), and the European Food Safety Authority (EFSA) [11,12,13], localized outbreaks have been traced back to foods and food packaging as the common point source, through the use of whole-genome sequencing [14,15]. Reports of SARS-CoV-2 being detected in dairy products, such as ice cream, raise concerns about virus-contaminated food and beverages being potential sources of infection [16]. The virus remains infectious on a variety of foods for up to 21 days, with the time of virus survival extended at low temperatures [14,17,18,19,20]. Proteins, fats, and moisture content appear to contribute to stabilization of infectious virus on foods, suggesting that beverages containing proteins and fats may also be capable of maintaining high concentrations of infectious virus [17].

SARS-CoV-2 mediates viral entry by binding to the angiotensin-converting enzyme 2 (ACE2), which is abundantly expressed in respiratory tissue, mediating viral entry in the respiratory tract [21,22]. However, the ACE2 receptor is also highly expressed in the oral cavity and gastrointestinal tract [23]. Therefore, SARS-CoV-2 in food or beverages may be able to enter the host through ingestion of contaminated foods or beverages. Given that a single cough from COVID-19 patients can deposit more than 100,000 viral particles carried in their respiratory fluid [7], and saliva may contain up to 100 million copies of virus [8], it is not unrealistic to consider that beverages may be contaminated by an individual shedding infectious SARS-CoV-2, promoting transmission if the beverage were to be shared with another individual. Therefore, in this study, we assessed the survival of infectious SARS-CoV-2 in common beverages, including non-alcoholic and alcoholic beverages, to determine the potential risks of infection from virus-contaminated beverages.

## 2. Materials and Methods

### 2.1. Cells and Viruses

SARS-CoV-2 (Isolate USA-WA1/2020, BEI NR-52281) was propagated and titrated on Vero-E6 cells (ATCC CRL-1586). Virus aliquots were thawed and maintained in a cold block during inoculation of beverages. Second passage virus stock was used for these studies.

### 2.2. Sample Preparation and Inoculation

All beverages were purchased from a local vendor, maintaining identical brands for each experiment. Beverage selections were based on popular brands of beverage consumption in the U.S. Dulbecco’s Modified Eagle’s Medium (DMEM) was used as a negative control and is referred to as “media” in the text and figures (Fisher Scientific, Waltham, MA, USA). Beverage samples (1 mL) were transferred into microcentrifuge tubes in duplicate for inoculation. Virus (1 × 10^4^ plaque-forming units, PFU) was transferred into each sample and vortexed briefly. Fifty microliters (50 μL) of each sample were immediately transferred into 450 μL DMEM (Fisher Scientific, Waltham, MA, USA) and vortexed (0 h sample). Two additional 10-fold dilutions were performed immediately, and all three dilutions were promptly transferred into plaque titer plates seeded with Vero E6 cells for plaque assay. One hour later, 50 μL of each sample were transferred into 450 μL of DMEM and 10-fold dilutions and plaque assays were performed for the 1 h time point. Some samples (milks, cocoa, media, and water) were placed into a 4 °C refrigerator for seven days and the same sampling procedure was followed to obtain 7 d samples. Each beverage experiment was repeated three times in duplicate.

### 2.3. Plaque Assay

To quantify the titer of infectious virus in beverage samples, a standard plaque assay was performed on each sample in duplicate. Serial dilutions of the beverage samples were inoculated onto confluent Vero E6 monolayers in 24 well plates in duplicate. The sample was incubated for 1 h to adsorb virus and then replaced with DMEM containing 8% fetal bovine serum (FBS), 1% penicillin/streptomycin (PS), and 0.5% agarose to limit the spread of the virus. The inoculum was back-titrated by plaque assay, in triplicate, to verify inoculum concentration for each experiment. The infected plates were incubated at 37 °C with 5% CO_2_ for 48 h, followed by fixation with formaldehyde and staining with plaque dye (60% methanol, 25% water, 10% formaldehyde, 5% acetic acid, 1% lyophilized crystal violet). Plaques were counted after the plates were dried and the results were expressed as plaque-forming units per mL (PFU/mL) recovered for each sample.

### 2.4. Viral Genome Extraction and RT-qPCR

In addition to the plaque titration, viral genome copies were quantified by qRT-PCR for the different concentrations of cocoa samples (10%, 5%, and 2.5%). At the same time as sample collection for plaque titration, 200 μL of the cocoa samples were transferred into 200 μL LS-Trizol (Fisher Scientific, Waltham, MA, USA). RNA was extracted from the samples using the Zymo RNA Micro-Prep Kit (ZYMO Research, Irvine, CA, USA), according to the manufacturer’s recommended protocol. To limit sedimentation in the extraction columns, cocoa samples were first centrifuged at 3000 rpm for 5 min to pellet cocoa solids before transferring the liquid to the Zymo spin columns. To control for potential binding and/or inhibition of the cocoa solids to the virus, uninoculated cocoa samples were centrifuged, 200 μL of the supernatant were transferred into 200 μL LS-Trizol and spiked with purified SARS-CoV-2 RNA. Two hundred microliters of water were also transferred to 200 μL LS-Trizol and spiked with the same volume of purified SARS-CoV-2 RNA as a control. To determine RNA genome copy number, 10 μL RT-qPCR reactions specific for the nucleocapsid gene of SARS-CoV-2 using the iTaq Universal Probe One-Step Kit (BioRad, Hercules, CA, USA) were run on a ViiA 7 Real-Time PCR system (Applied Biosystems, Foster City, CA, USA), as described previously [17]. The standard setting was used for the reaction with the following cycle conditions: 1 cycle of 10 min at 50 °C followed by 2 min at 95 °C; and 45 cycles of 3 s at 95 °C and 30 s at 55 °C. Results were reported as genome copy number per mL of beverage to allow for direct comparison to infectious viral titer measured in PFU/mL.

### 2.5. Statistical Analysis

All experiments were performed three times, in duplicate, with freshly purchased and opened beverages each time. Plaque assay data were converted to log PFU/mL prior to statistical analysis. Statistical analyses were conducted in JMP (SAS Institute, Cary, NC, USA), using analysis of variance (ANOVA) followed by Dunnett’s post hoc test for comparison of 0 h and inoculum, Tukey’s HSD for comparison of beverages with 0 h, 1 h, and 7 d time points, and paired *t*-test for comparison of beverages with 0 h and 1 h time points. Significant differences were detected at *p* < 0.05. Error bars = SEM. Correlations were analyzed using bivariate analysis in JMP.

## 3. Results

We previously determined that SARS-CoV-2 is able to remain infectious for up to 21 days on a variety of different foods [17,20]. In those studies, we determined that certain types of foods, including meats and seafoods, support a longer survival time of the virus on their surface compared to fruits and vegetables, many of which have antiviral properties. These findings suggested that proteins and fats combined with a high moisture content provide a stabilizing effect on maintaining the infectivity of the virus. Therefore, we sought to assess the capabilities of several different categories of common beverages to support the survival of infectious SARS-CoV-2, if they were to become contaminated with the virus. We initially screened several different beverages and then expanded each category based on our preliminary results.

### 3.1. Milk

**Figure 1:**
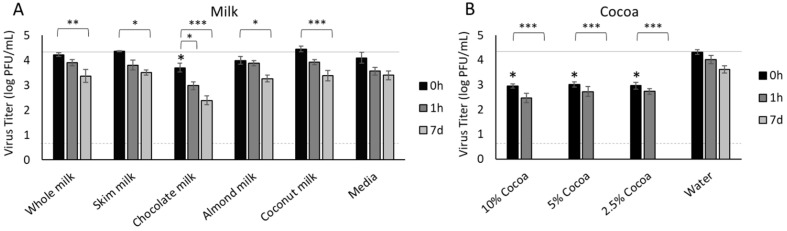
Survival of SARS-CoV-2 in milk and cocoa. SARS-CoV-2 was recovered from bovine- and plant-based milk products (**A**) and different concentrations of cocoa dissolved in water (**B**) immediately after inoculation (0 h) and at 1 h and 24 h after inoculation. Infectious virus was quantified by plaque assay on Vero E6 cells and shown as log PFU/mL. No significant differences were detected between inoculum and 0 h for any of the milk products except chocolate milk (*p* = 0.0122). 10%, 5%, and 2.5% cocoa were significantly reduced at 0 h compared with the inoculum (*p* < 0.0001). The inoculum is shown as a solid gray line (4.3 log PFU/mL) and the detection limit of the plaque assay is shown as a dashed line (0.7 log PFU/mL). Significant differences between 0 h and inoculum are shown with an asterisk. Significant differences between means at 0 h and later time points are shown by brackets and asterisks (* <0.05; ** <0.01; *** <0.001). Error bars = SEM.

In our initial screen, we determined that SARS-CoV-2 remained infectious in whole milk for one hour with minimal reduction in infectious virus titer. In our previous studies with foods [17,20], fats appeared to provide a stabilizing effect on SARS-CoV-2 and plant-based foods were often detrimental to virus survival. Therefore, we assessed SARS-CoV-2 survival in whole milk (3.25% fat), skim milk (0% fat), chocolate milk (1% fat), almond milk (1% fat), and coconut milk (1.9% fat), providing a broad sampling of fat content and plant-based milk alternatives. With the exception of chocolate milk, SARS-CoV-2 remained infectious in all of the milks for seven days, with minimal reduction in virus titer at 1 h post-inoculation (hpi) and loss of less than 1 log PFU/mL by 7 dpi (4.3 log PFU/mL at 0 h reduced to 3.3–3.5 PFU/mL at 7 d) (Figure 1A). In chocolate milk, the infectious virus titer was reduced 94.5% within 1 h post-inoculation (4.3 log PFU inoculum reduced to 3.0 log PFU/mL at 1 hpi) and 98.5% by 7 dpi (further reduced to 2.4 log PFU/mL). Since the primary difference between chocolate milk and the other milks was the inclusion of cocoa, we then tested a panel of cocoa dissolved in water at different concentrations. Compared to water, cocoa significantly reduced the infectivity of SARS-CoV-2 immediately after inoculation (0 h) from 4.3 log PFU inoculum to 3.0 log PFU/mL (all concentrations, *p* < 0.0001) (Figure 1B). All three concentrations of the cocoa (10%, 5%, 2.5%) further reduced the infectious virus titer to 2.5–2.7 log PFU/mL within 1 h and fully inactivated the virus by 7 dpi (*p* < 0.0001 compared to 0 h) (Figure 1B).

To determine if cocoa may degrade the viral RNA in addition to reducing virus infectivity, we also assessed SARS-CoV-2 RNA genome copies in the cocoa samples. We detected no SARS-CoV-2 RNA in the cocoa samples, regardless of cocoa concentration, demonstrating that cocoa can reduce viral infectivity and also degrade the viral RNA. As a control, we prepared 10%, 5%, and 2.5% cocoa in water and spiked the samples with purified SARS-CoV-2 RNA, which also failed to amplify by qRT-PCR assay. Control samples of water with infectious virus, as well as water spiked with purified SARS-CoV-2 RNA, amplified the SARS-CoV-2 genome as expected.

### 3.2. Coffee, Tea, and Soda

**Figure 2:**
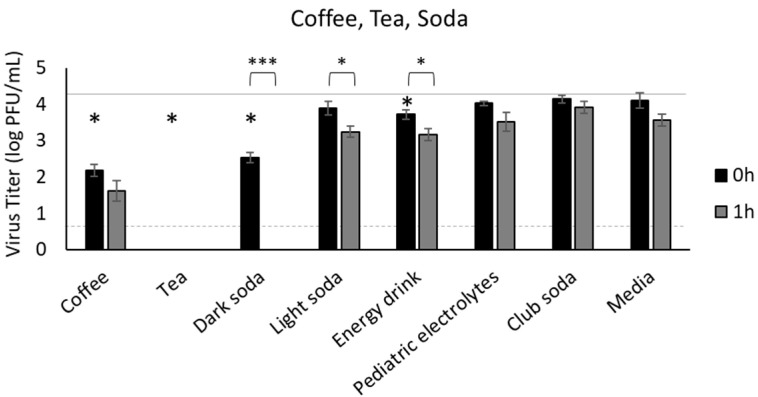
Survival of SARS-CoV-2 in coffee, tea, soda, energy drink, and pediatric electrolyte beverage. SARS-CoV-2 was recovered from beverages immediately after inoculation (0 h) and 1 h later, quantified by plaque assay on Vero E6 cells and shown as log PFU/mL. Infectious virus was significantly reduced at 0 h in coffee, tea, dark soda (*p* < 0.0001), and energy drink (*p* < 0.0177). The inoculum is shown as a solid gray line (4.3 log PFU/mL) and the detection limit of the plaque assay is shown as a dashed line (0.7 log PFU/mL). Significant differences between 0 h and inoculum are shown with an asterisk. Significant differences between means at 0 h and 1 h are shown by brackets and asterisks (* <0.05; *** <0.001). Error bars = SEM.

Coffee reduced SARS-CoV-2 infectivity 99% immediately after inoculation of the virus into the beverage (from 4.3 PFU/mL to 2.18 PFU/mL at 0 h, *p* < 0.0001 compared to inoculum) and further reduced viral titer one hour later (to 1.62 PFU/mL at 1 h) (Figure 2). However, we were unable to recover infectious virus from black tea at either time point (*p* < 0.0001 compared to inoculum). The infectious virus titer was significantly reduced in dark soda immediately after transferring the virus into the beverage (from 4.3 PFU/mL to 2.52 PFU/mL at 0 h, *p* < 0.0001), and we were unable to detect infectious virus one hour later (Figure 2). Although the infectious virus titer was significantly reduced in light soda and energy drink by 1 hpi (*p* = 0.029 and 0.034, respectively), these two beverages were still able to maintain 3.2 log PFU/mL infectious SARS-CoV-2 (Figure 2). In contrast, we detected minimal reduction in infectious virus in a pediatric electrolyte beverage or in club soda, which were both comparable to infectious virus in media (Figure 2).

### 3.3. Beer

**Figure 3:**
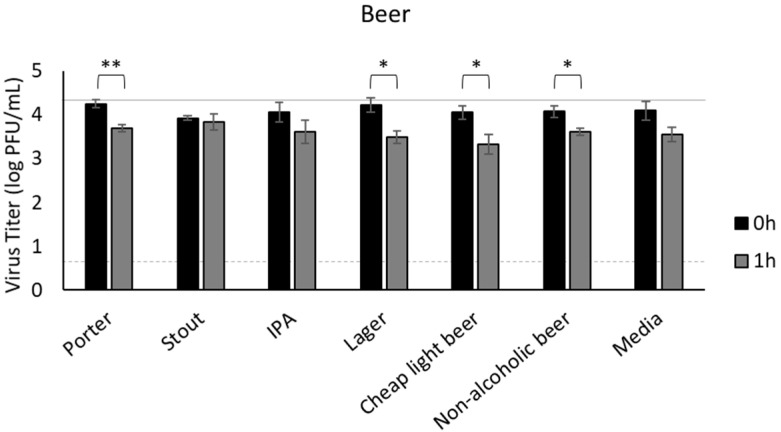
Survival of SARS-CoV-2 in beer. SARS-CoV-2 was recovered from beverages immediately after inoculation (0 h) and 1 h later, quantified by plaque assay on Vero E6 cells, and shown as log PFU/mL. The inoculum is shown as a solid gray line (4.3 log PFU/mL) and the detection limit of the plaque assay is shown as a dashed line (0.7 log PFU/mL). Significant differences between means at 0 h and 1 h are shown by brackets and asterisks (* <0.05; ** <0.01). Error bars = SEM.

We next wanted to test beer, as this beverage is popular among individuals who frequent crowded social venues. If beer supports survival of SARS-CoV-2, sharing of contaminated beverages could potentially compound the risk of infection due to respiratory transmission in these venues. Despite testing a broad selection of beers, infectious virus was maintained at high titer for at least one hour after inoculation into the beverages (Figure 3). Although porter, lager, and cheap light beer did produce a statistically significant reduction in viral titer after 1 h (*p* < 0.05 at 1 h), these three types of beer still retained 3.3–3.7 log PFU/mL of virus one hour post-inoculation. Recovery of infectious virus from non-alcoholic beer was similar to the other beers, all of which contained 4.2–6.9% alcohol, demonstrating that this concentration of alcohol has negligible effects on SARS-CoV-2 infectivity in one hour.

### 3.4. Hard Cider, Wine and Fruit Juices

**Figure 4:**
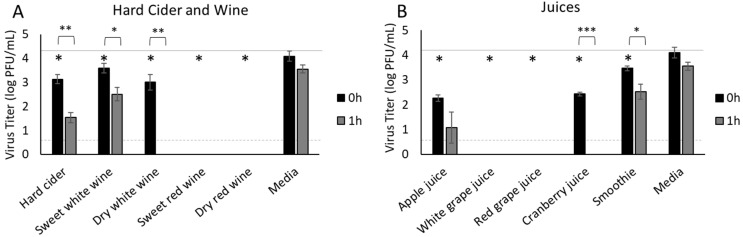
Survival of SARS-CoV-2 in hard cider, wine, and fruit juices. SARS-CoV-2 was recovered from hard cider and wine (**A**) and fruit juices (**B**) immediately after inoculation (0 h) and 1 h later. Infectious virus was quantified by plaque assay on Vero E6 cells and shown as log PFU/mL. All beverages significantly reduced infectious virus immediately after inoculation at 0 h compared to inoculum (*p* < 0.0001). The inoculum is shown as a solid gray line (4.3 log PFU/mL) and the detection limit of the plaque assay is shown as a dashed line (0.7 log PFU/mL). Significant differences between 0 h and inoculum are shown with an asterisk. Significant differences between means at 0 h and 1 h are shown by brackets and asterisks (* <0.05; ** <0.01; *** <0.001). Error bars = SEM.

Next, we sought to determine if SARS-CoV-2 could survive in hard cider and wine, which are produced from fermentation of fruits. We previously found that some fruits have an antiviral effect on SARS-CoV-2. Hard cider produced an immediate significant reduction in the infectious virus titer compared to the inoculum (4.3 PFU/mL inoculum reduced to 3.13 PFU/mL at 0 h, *p* < 0.0001) and further reduced the infectious virus to 1.53 log PFU/mL by 1 hpi (*p* = 0.0027 compared to 0 h; Figure 4A). Immediately after inoculation (0 h), we recovered 3.59 log PFU/mL and 3.01 log PFU/mL of virus from sweet and dry white wine, respectively (Figure 4A). One hour later, we recovered 2.51 log PFU/mL from sweet white wine, but the dry white wine had fully inactivated the virus (Figure 4A). However, we were unable to recover infectious virus from red wine, either sweet or dry, at either time point.

To determine if these effects on the virus infectivity were due to the fermentation process and alcohol content or to inherent characteristics of the fruit juices used to produce hard cider and wine, we then tested apple juice and both white and red grape juices. Apple juice reduced infectious virus from 4.3 log PFU/mL inoculated into the juice to 2.26 log PFU/mL, representing a 99% reduction in viral titer immediately after inoculation (*p* < 0.0001 at 0 h compared to inoculum, Figure 4B), and further reduced viral titer to 1.08 log PFU/mL one hour later. Thus, the effects of apple juice on infectious SARS-CoV-2 were similar to hard cider, which contains 5% alcohol. Infectious SARS-CoV-2 could not be recovered from either white or red grape juice at either 0 h or 1 h time points (Figure 4B), suggesting that inherent components of grape juice can inactivate SARS-CoV-2. To determine if other types of fruit juices had similar effects on SARS-CoV-2, we also tested cranberry juice and a fresh fruit smoothie made from mango, strawberries, pineapple, and orange juice. Cranberry juice significantly reduced the infectious viral titer at 0 h (from 4.3 log PFU/mL inoculum to 2.44 log PFU/mL, *p* < 0.0001) and infectious virus was not detectable by 1 hpi (*p* < 0.0001 compared to 0 h). Surprisingly, the fresh fruit smoothie supported infectious virus the best of all of the juices we tested, reducing the infectious viral titer from 4.3 log PFU/mL inoculum to 2.52 log PFU/mL at 1 h; this reduction was, however, a significant difference from 0 h (3.47 log PFU/mL; *p* = 0.0005).

### 3.5. Liquor

**Figure 5:**
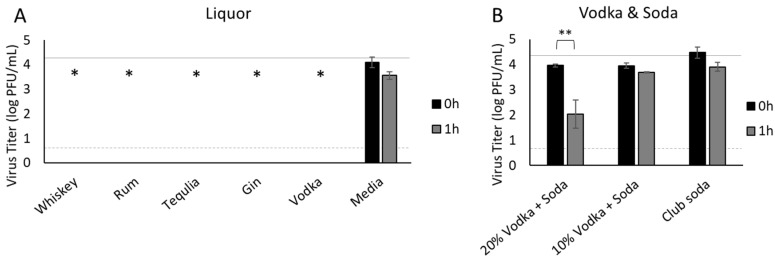
Survival of SARS-CoV-2 in liquor. SARS-CoV-2 was recovered from liquors (**A**) and mixed beverages consisting of vodka and club soda to different alcohol percentages (**B**) immediately after inoculation (0 h) and 1 h later, quantified by plaque assay on Vero E6 cells and shown as log PFU/mL. Infectious virus was significantly reduced immediately after inoculation (0 h) in liquors (*p* < 0.0001). The inoculum is shown as a solid gray line (4.3 log PFU/mL) and the detection limit of the plaque assay is shown as a dashed line (0.7 log PFU/mL). Significant differences between 0 h and inoculum are shown with an asterisk. Significant differences between means at 0 h and 1 h are shown by brackets and asterisks (* <0.05; ** <0.01). Error bars = SEM.

Since we found minimal detrimental effects of alcohol content on infectious virus from beer with up to 6.9% alcohol content, and the detrimental effects of hard cider (5% alcohol) and wine (10–14.7% alcohol) appear to be attributable to inherent effects of the fruit juices from which they are produced rather than the alcohol, we also assessed hard liquors. We included whiskey, rum, tequila, gin, and vodka, selecting brands that all contained 40% alcohol. As expected, no infectious virus was recovered from any of these beverages (Figure 5A), demonstrating that a critical threshold of alcohol content had been reached at 40% alcohol. Considering that many people do not drink liquor straight, we also tested simulated cocktails, which would have a lower alcohol percentage when the liquor is mixed with a non-alcoholic beverage. To avoid confounding effects of juices or sodas, we mixed vodka with club soda, which had no significant antiviral effect up to 1 h (Figure 2 and shown in Figure 5B for comparison), to achieve simulated cocktails with 20% (1:1 mixture vodka and club soda) and 10% (1:2 mixture) alcohol content. Although vodka and soda with 20% alcohol significantly reduced infectious virus by 1 h post-inoculation (*p* = 0.037), no significant reduction was observed for the mixed drink with 10% alcohol over the same time period (*p* > 0.05, Figure 5B).

### 3.6. Characteristics of Beverages

**Figure 6:**
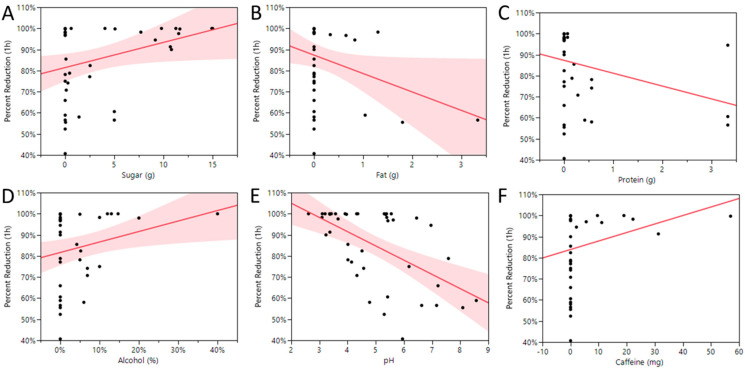
Correlations between beverage characteristics and percent reduction in virus titer at 1 h. Higher concentrations of sugar (**A**) and alcohol (**D**) correlate with greater reduction in virus titer by 1 h post-inoculation (*p* = 0.0378, and 0.0189, respectively). Increased percentage of fat (**B**) and higher pH (**E**) correlate with less reduction in virus titer by 1 h post-inoculation (*p* = 0.0478 and <0.0001, respectively. Shaded areas = confidence intervals (0.05). No correlation was found between protein (**C**) or caffeine (**F**) content and reduction in virus titer by 1 h post-inoculation (*p* > 0.05). Bivariate analysis, JMP.

The beverages we tested contained a wide range of sugar (0–15 g/100 mL), fat (0–3.33 g/100 mL), protein (0–3.33 g/100 mL), caffeine (0–57 mg/100 mL), and alcohol (0–40%) content. The pH also varied widely (2.61–8.57). These characteristics are shown in Table 1. To determine if any of these characteristics had a significant effect on reducing infectious virus, we analyzed the percent reduction in the infectious virus titer at 1 h compared with each of the characteristics by bivariate analysis (Figure 6). We identified significant effects of sugar and alcohol, with higher concentrations of sugar and alcohol correlating with greater reductions in SARS-CoV-2 infectivity (Figure 6A,D, *p* = 0.0378 and 0.0189, respectively). We also identified a weak, yet statistically significant, correlation between fat concentration and reduction in viral titer, with lower concentrations of fat correlating with greater reductions of infectious virus (*p* = 0.0478, Figure 6B). Our strongest correlation was between pH and reduction in infectious virus, with lower pH correlating with greater reduction (*p* < 0.0001, Figure 6E). However, infectious virus was reduced 100% within one hour in beverages ranging from pH 2.61 to 5.55, while beverages with pH as low as 4.78 were among the weakest at neutralizing the infectious virus titer, demonstrating that other characteristics of the beverages also contributed substantially to inactivation or protection of virus viability. We found no correlations between protein or caffeine content and reduction in infectious virus in the beverages we tested, although the majority of these beverages contained no protein or caffeine (Figure 6C,F). Although we found statistically significant correlations between sugar, alcohol, and fat content, as well as pH, other characteristics and components of many of these beverages likely impacted these correlations.

### 3.7. Saliva

**Figure 7:**
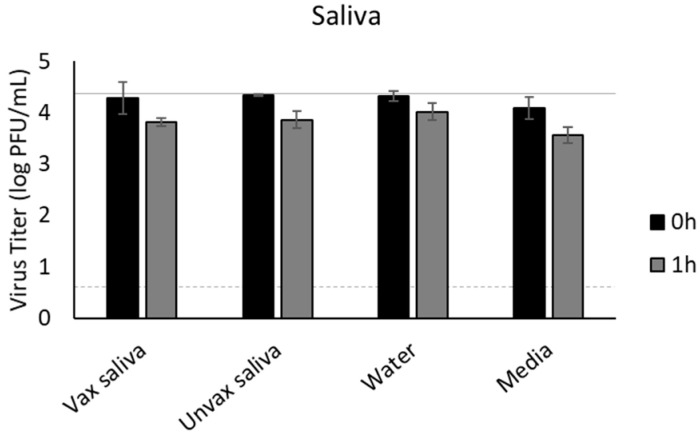
Survival of SARS-CoV-2 in saliva from vaccinated (vax saliva) and unvaccinated (Unvax saliva) individuals compared to water and media controls. SARS-CoV-2 was recovered immediately after inoculation (0 h) and at 1 h and 7 d after inoculation, quantified by plaque assay on Vero E6 cells, and shown as log PFU/mL. The inoculum is shown as a solid gray line (4.3 log PFU/mL) and the detection limit of the plaque assay is shown as a dashed line (0.7 log PFU/mL). No statistically significant differences were identified. Error bars = SEM.

Beverages would likely be contaminated through virus carriage in saliva, which contains a variety of antimicrobial properties. Therefore, we also assessed the survival of SARS-CoV-2 in natural saliva. Since the currently approved mRNA vaccines have been shown to elicit secretory IgA (sIgA) antibodies in the saliva of ≈60% of vaccinees [24], we included saliva from both vaccinated and unvaccinated individuals. Saliva, from either unvaccinated or vaccinated individuals, did not significantly reduce the infectivity of SARS-CoV-2, with results similar to water and media (Figure 7).

## 4. Discussion

SARS-CoV-2 infectious virus has been isolated from the saliva of symptomatic COVID-19 patients up to 15 days after symptom onset and viral RNA has been detected up to 25 days after symptoms have appeared [8,9,25,26]. Similarly, viral RNA and infectious virus have been recovered from the saliva of mildly symptomatic and asymptomatic people infected with SARS-CoV-2 [10,27,28]. It is not surprising that infectious SARS-CoV-2 can be detected in saliva, as numerous viruses that replicate in the oral or nasal epithelium are capable of being shed in and transmitted by saliva, including herpes simplex viruses (HSV), Epstein-Barr virus, human papillomavirus, hepatitis C virus (HCV), norovirus, and rabies [29]. As the response to the pandemic continues to be variable from locale to locale, with some locations reopening (or never having closed) social gathering points such as bars, restaurants, and night clubs with variable requirements for masking and social distancing, outbreaks of COVID-19 continue to be associated with these venues [30,31,32]. Even when such venues are closed, people continue to host and attend large social gatherings, where masking and distancing are not, or cannot be, enforced. While transmission has been viewed as being driven by respiratory droplets and aerosols in these settings, it is worth investigating whether or not infectious virus can survive in the beverages consumed and shared among patrons of such establishments and social events, as they have been indicated as potential point sources for transmission [33]. In private family settings, if one member of the family is shedding SARS-CoV-2, the potential exists for contamination of beverages within the household, such as in milk, juice, or soda. It is apparent that SARS-CoV-2 is capable of infecting epithelial cells in the oral mucosa and salivary glands, as viral entry factors and viral RNA have been detected in both [27,34]. These findings suggest that transmission of SARS-CoV-2 via saliva may be an underappreciated mode of viral transmission. Therefore, our goal was to determine if beverages could, indeed, present a risk of infection if they were to become contaminated with SARS-CoV-2. The risk of contamination during manufacture and processing of the beverages we tested is minimal, as these products are typically processed in closed systems, rendering virus ingress highly unlikely. Thus, our main focus was at the level of the consumer, who may share beverages during social gatherings or within a household.

Since SARS-CoV-2 can be shed in saliva, the question is raised as to whether it can infect the gastrointestinal (GI) tract of those exposed. GI tract symptoms are commonly reported among symptomatic COVID-19 patients and up to half of those infected shed viral RNA in their stool, including up to 39% of asymptomatic individuals [9,35,36]. While it is unclear if this RNA represents productive viral infection along the GI tract, the SARS-CoV-2 receptor (ACE2) is expressed in tissues of the GI tract, and SARS-CoV-2 nucleocapsid has been detected in epithelial cells of the stomach, small intestine, and rectum of hospitalized COVID-19 patients [35]. Although the acidity of the stomach is typically lower than pH 3.5, which may inactivate SARS-CoV-2, simply drinking plain water (200 mL) can increase gastric pH to >4 [37]. Considering SARS-CoV-2 infectivity is retained in a wide range of pH values at room temperature (pH 3–10) [38], the virus may survive in the gastric environment with a drink. Direct intragastric challenge with SARS-CoV-2 of rhesus macaques resulted in productive infection along the GI tract as well as pulmonary disease, indicating that SARS-CoV-2 can disseminate to other tissues in the host following GI tract infection [39]. Combined, these data suggest that the GI tract may support infection of SARS-CoV-2 and may represent an alternative route of infection in some patients.

SARS-CoV-2 can spread from infected patients to food and food packaging and remain infectious for up to 21 days [17,20,40]. Virus absorption to food products is driven by establishing strong hydrogen bonds between water molecules and the virus spike protein [41]. Since beverages are primarily composed of water, it is not surprising that the virus might form such bonds in beverages. Nonetheless, each of the beverages we tested has unique characteristics (e.g., pH, fat, sugar, and alcohol content) that may influence the stability of the virus. Some of the beverages we tested showed a minimal reduction in infectious SARS-CoV-2, which may present a risk of infection. Other beverages, however, naturally contain antiviral components, while others likely have additives that destabilize or inactivate SARS-CoV-2.

While the overall consumption of cow’s milk has been steadily declining since the 1940s in the U.S., Americans on average still consume ≈17.3 gallons of milk per person per year [42]. Given that cow’s milk is still commonly consumed across all age groups, we sought to determine the impact on the recovery of infectious SARS-CoV-2 in milk. Thermal processing (75 °C for 15–60 min) is one of the most effective methods to inactivate SARS-CoV-2 [43] and in full-fat milk, high-temperature short-time-pasteurization significantly reduces the concentration of SARS-CoV-2 if it were to be contaminated prior to pasteurization [44]. Within a household, however, virus shed in saliva may contaminate a common source, if, for example, members of the household were to drink from the jug. Since whole milk (with sales of 15,534 million pounds in 2020) and skim milk (with sales of 2845 million pounds in 2020) are among the most commonly sold fluid milk products in the U.S., we selected both whole milk and skim milk for analysis to span the range of milk fat content [45]. Infectious SARS-CoV-2 was recovered from both milk types at comparable levels across all time points, demonstrating that fat concentration did not substantially impact the recovery of infectious virus from these two milk products. As these are unflavored milks, we also included chocolate milk in our analysis, as 765 million pounds of flavored cow’s milk were sold in the U.S. in 2020 and it is popular among children [45]. Interestingly, there was a significant reduction in the infectious viral titer in chocolate milk at 7 days post inoculation, suggesting that the presence of cocoa may be influencing the stability of the virus. Cocoa, in various concentrations from 2.5% to 10% in water, showed a strong antiviral effect on SARS-CoV-2, destroying both viral infectivity and the RNA viral genome. Cocoa has been shown to have a similar dose-dependent inhibition of viral infectivity against other respiratory viruses including influenza A (H1N1, H3N2), influenza B, and avian influenza (H5N1, H5N9) in vitro. Cocoa has also shown antiviral properties in vivo, as mice infected with a lethal dose of influenza virus were protected following treatment with cocoa extract [46]. Additionally, recent in silico modeling studies suggest that flavan-3-ols and dimeric proanthocyanidins in cocoa are capable of binding to and interfering with the function of the main protease of SARS-CoV-2, which was verified through in vitro protease inhibition studies [47]. Having assessed the impact on recovery of infectious virus in cow’s milk, we then turned our attention to plant-based analogs of milk as their popularity and variety have steadily increased over time, representing ≈10% of the milk/non-dairy milk sales in the U.S. [42]. Infectious virus was recovered from both almond and coconut milk at comparable rates to cow’s milk. Thus, recovery of infectious virus across all milk product types was not influenced by fat content, pH, or dairy vs. non-dairy origin. The most intriguing result from this selection of beverages is the antiviral properties displayed by cocoa, which merit further study.

Since milk, both dairy and non-dairy, accounts for only ≈5.5% of the daily fluid intake for adults in the U.S. [48], we also assessed the impact of other popular beverages including coffee, tea, soda, and energy drinks on the recovery of infectious SARS-CoV-2. Coffee, which accounts for ≈14.9% of the daily fluid intake for adults in the U.S., had a significant antiviral effect against SARS-CoV-2. Coffee extracts have previously been shown to have antiviral activity against HSV, which is another enveloped virus, through both direct inactivation of the virus particle and inhibition of progeny formation [49]. While we did not assess the effects of coffee on progeny formation, the loss of infectivity in our assays was apparent, as greater than 99% of infectious SARS-CoV-2 was inactivated immediately following inoculation. Coffee extracts also inhibit replication of poliovirus, a non-enveloped RNA virus, suggesting that coffee may also impact the viral genome [49]. Tea accounts for ≈8.7% of the daily fluid intake for adults in the U.S. [48] and was found to completely ablate infectious virus immediately upon inoculation, suggesting a profound antiviral effect. The antiviral activity of black tea, likely mediated by theaflavins, is well documented against Sindbis Virus, influenza A, influenza B, HSV-1, HSV-2, bovine rotavirus, HCV, human immunodeficiency virus 1, and bovine coronavirus [50]. In silico molecular docking studies indicate that theaflavins in black tea can bind to the receptor-binding domain of the SARS-CoV-2 spike protein, the RNA-dependent RNA polymerase, and the main protease, indicating multiple points at which the infection cycle of SARS-CoV-2 can be blocked or interrupted, including binding and entry, genome replication, and progeny assembly [50]. In vitro infectivity assays and downstream RT-qPCR and Western blot analysis of SARS-CoV-2 incubated with tea extract rich in theaflavins showed that viral infectivity was ablated, viral RNA was destroyed, and structural changes were introduced in the S2 subunit of the spike protein [51]. SARS-CoV-2 in saliva was inactivated within 10 s after treatment with black tea and green tea, respectively [52]. Considering our studies show no significant reduction of SARS-CoV-2 in saliva alone, consumption of tea while shedding virus may be beneficial.

Soda and energy drinks account for ≈10.2% of the daily fluid intake for adults in the U.S. (19.9% for children) [48,53]. In our studies, we found variable antiviral activity based on soda type (dark vs. light). Dark soda, like coffee, had a noted reduction in recovery of infectious virus immediately following inoculation. Unlike coffee, dark soda completely inactivated SARS-CoV-2 within one hour. Light soda and an energy drink failed to produce similar reductions. This suggests a potential antiviral effect of some unknown compound in dark soda. A number of confounding variables including temperature, caffeine content, carbonation, and pH should be considered when interpreting these results. The reduction in infectious virus was not temperature mediated, as all beverages were at the same temperature at the time of inoculation. Although caffeine was reported to play a small role in antiviral activity of coffee extract against HSV [49], the coffee in our studies had a much greater antiviral effect than the energy drink, although these two beverages had similar caffeine content. It is worth noting that infectious virus was recovered at comparable levels from both the caffeine-free light soda and the highly caffeinated energy drink, suggesting caffeine content likely had minimal impacts. Similarly, carbonation was not a driver of reduced infectious virus recovery as carbonated club soda showed the least reduction among all beverages used, producing results similar to plain media and water. Finally, reduction in infectious virus is independent of low pH in these beverages as pH ranged from 2.6 (dark soda) to 5.3 (coffee and tea) with varying levels of inactivation, including immediate inactivation of virus in tea but not coffee, which had the same pH. Although we did find statistically significant correlations between pH and reduction in infectious virus, the pH range at which virus was fully inactivated within one hour overlapped with the range of pH of the beverages with the weakest antiviral effects. While pH does play a role, other components likely have a greater effect on the virus than the acidity or alkalinity of the beverages that we tested.

Juices, including smoothies, account for ≈5.6% of the daily fluid intake for adults in the U.S. (7.3% for children) [48,53]. All of the juices we tested had profound antiviral effects on SARS-CoV-2. In our previous studies, we found just the apple skin to have antiviral properties against HSV-1, which increased if the apple skin and pulp were macerated together, suggesting that the apple juice had greater antiviral effects than the skin [17]. We did not, however, see a similar antiviral effect of apple skin on SARS-CoV-2 [17]. Apple juice, the least effective against SARS-CoV-2 in our studies, has been shown to exert antiviral effects against both poliovirus type 1 (PV1) and coxsackievirus type B5 (CV-B5) in in vitro infectivity assays, although no specific mechanism was proposed [54]. Extracts of apple pomace have been shown to inhibit HSV-1 and HSV-2 replication in cell culture, likely by interfering with viral binding and entry [55]. Cranberry juice showed comparable inhibition at 0 h but completely inactivated SARS-CoV-2 within one hour. Long since known to inhibit bacterial cell adhesion through high molecular weight substances, cranberry juice was also shown in vitro to inhibit binding and entry of influenza A (H1N1, H3N2) and influenza B through interference with neuraminidase activity, thereby inhibiting exit and release of viral progeny [56,57]. Capitalizing on these data, Oximacro, a virucidal therapeutic developed from cranberry extract enriched in A-type proanthocyanidins, prevented viral entry of influenza and HSV in vitro [58,59]. Thus, fruit-based products may be worth investigating for virucidal activity against SARS-CoV-2. Surprisingly, we detected minimal viral inhibition from the mixed fresh fruit smoothie. Given that nearly all of our results with food and beverages have shown that fresh produce has antiviral properties, we had expected the combination of fresh mango, pineapple, and strawberries blended with orange juice would exhibit antiviral properties, since mangos [60], pineapples [61], and orange juice [62] are rich in antioxidants and enzymes. In summary, antiviral activity against SARS-CoV-2 mediated via fruit juice and fruit juice-based beverages was observed in a fruit-dependent manner.

Having investigated the effects of beverages that constitute the majority of the daily fluid intake for adults in the U.S. on the recovery of infectious SARS-CoV-2, our attention turned to alcoholic beverages as ≈61.2% of U.S. adults classify themselves as light to moderate weekly drinkers [63]. We selected commonly available red and white wines, each with and without residual sugars (sweet and dry). Red wines, both sweet and dry, showed immediate ablation of infectivity, regardless of residual sugar content. Interestingly, immediate inactivation of infectious virus was not observed for white wine. Both sweet and dry white wines had comparable levels of infectious SARS-CoV-2 immediately after inoculation. Although the sweet white wine further reduced the infectious virus titer within one hour, no infectious virus could be recovered from the dry white wine at that time point. This is an intriguing observation as non-fermented white and red grape juices completely neutralized infectious SARS-CoV-2 immediately following inoculation, indicating significant antiviral activity. Red grape juice has previously been shown in vitro to inhibit replication of CV-B3, CV-B5, echovirus types 6 and 7, PV1, and HSV-1, by interruption of viral proteins by phenolic compounds [64,65]. Similar viral inhibition has been documented for white wine and red wine when incubated with echovirus type 7, HSV-1, PV1, and reovirus, with red wine producing a more potent effect on virus inactivation than did white wine [65]. This inhibition was proposed to be due to the increased presence of phenolic compounds in red wines compared to white wines. The increased concentration of phenolics occurs through prolonged contact between grape skins, grape seeds, and grape juice, which occurs during red wine production but not during white wine production [65]. One such phenol present in grape skins and red wine, resveratrol, has been shown through in vitro studies to inhibit HSV-1 and HSV-2 through suppression of early and late viral gene activation, thereby interfering with the production of critical viral proteins for progeny virus assembly [66]. However, since SARS-CoV-2 does not replicate or express viral genes in beverages, some other mechanism appears to inactivate this coronavirus. Additionally, in vitro studies demonstrated that resveratrol exhibits antiviral activity against Middle East respiratory syndrome coronavirus (MERS-CoV), mediated by blocking viral entry [67], which is likely the more relevant mechanism to our studies. Of note, in silico analysis indicates that resveratrol may bind to and inhibit the main protease of SARS-CoV-2 [68]. In addition to resveratrol, tannin, a phenolic compound that is abundant in grape skin and seeds [69], was reported to inhibit SARS-CoV-2 through binding and inhibiting the main protease [70,71] and host transmembrane protease serine 2 [71]. In humans, consumption of wine has been reported to provide a protective effect against COVID-19, particularly red wine [72]. While interpreting these data, the impact of pH and alcohol concentration should be considered. The pH was fairly consistent across the wines, ranging from 3.0 (sweet white) to 3.4 (sweet red), refuting a possible pH effect. Some of the reduction in infectious virus recovery may be attributable to alcohol concentration, as it ranged from 10% (sweet white) to 14.7% (sweet red) and increasing alcohol concentration did correlate with increasing reduction in infectious virus. Although alcohol content may play some role, phenolic compounds are more likely to be responsible for the distinctive differences we observed between red and white wine, as these compounds are more abundant in red wine than in white wine.

Moving from wines to beer, a range of beers were chosen to represent the diversity available to the average consumer, including an inexpensive light beer, lagers, India pale ales, porters, and stouts. Non-alcoholic beer was also included as a control for any alcohol-mediated effects on virus stability. Although beer exerts antimicrobial properties independent of alcohol through substances derived from hops and phenolic acids, these effects have not been widely explored for viruses. For SARS-CoV-2, all beers, regardless of type, behaved similarly showing limited reduction in virus. This limited reduction (less than 1 log PFU/mL) was likely not attributable to pH as the pH was fairly consistent across the beers ranging from 4.0 (light beer) to 4.7 (stout). Alcohol percentage, which ranged from 4.2% (light beer) to 6.9% (porter and IPA), was also likely not responsible, as the same reduction was observed in the non-alcoholic beer. Considering that all of the beers still contained at least 3.3 log PFU/mL virus an hour after inoculation, and infectious SARS-CoV-2 can be shed in saliva even when asymptomatic, sharing of beer should be considered as posing a potential risk of infection during social events. Indeed, consumption of beer and hard cider increased the risk of SARS-CoV-2 infection in a large population-based analysis in the United Kingdom, regardless of frequency and amount of alcohol intake [72].

The higher alcohol content in liquors, which were all 40% alcohol by volume, was sufficient to fully inactivate SARS-CoV-2 immediately following inoculation of whiskey, rum, tequila, gin, and vodka. This indicates a critical threshold of alcohol concentration had been reached. While some patrons consume their liquor unadulterated by other beverages, the question should be addressed as to whether the addition of other non-alcoholic beverages into a liquor to produce a cocktail with a lower alcohol concentration may impact the recovery of infectious SARS-CoV-2. To this end, vodka (40% alcohol) was diluted to achieve mixed drinks with 20% and 10% alcohol, selecting club soda since liquors are commonly mixed with sodas and our data indicate that club soda does not, by itself, significantly affect the survival of SARS-CoV-2. While the vodka and soda containing 20% alcohol inactivated 98% of the virus within an hour, the mixed drink containing 10% alcohol did not significantly reduce infectious virus in the same time period, demonstrating that mixing liquors with other non-alcoholic beverages substantially reduces the capacity of the alcohol content to inactivate SARS-CoV-2.

Given that both non-alcoholic and alcoholic beverages are sometimes shared amongst individuals, which poses a risk of introduction of saliva into the beverage, we also determined the impact of saliva on the recovery of infectious SARS-CoV-2. Although saliva contains antimicrobial agents, it did not significantly reduce infectivity of SARS-CoV-2 within one hour. Furthermore, we found no differences between saliva from vaccinated and unvaccinated individuals. This is not necessarily surprising as intramuscular administration of vaccines is not known to induce strong secretory IgA (sIgA) production, which would be the most likely antibody responsible for providing mucosal protection against SARS-CoV-2 [73]. While the currently approved mRNA vaccines have been shown to elicit sIgA in the saliva of ≈60% of vaccinees, viral infectivity inhibition studies have not been performed to demonstrate the neutralization capacity of these antibodies [24]. Given the small sample size of vaccinated and unvaccinated volunteers used in our study, no definitive conclusions should be drawn regarding the neutralization potential of sIgA present in the saliva of vaccinated individuals.

## 5. Conclusions

Taken together, our data show that infectious SARS-CoV-2 can be recovered from commonly consumed beverages in a beverage type and time-dependent manner. While we do not know if the level of infectious virus recovered from these beverages is enough to establish infection in the oropharynx, survive transit through the GI tract, establish infection in the GI tract, or disseminate through the host if local replication occurs at any of these points, these questions are worth addressing. Although aerosol or droplet transmission is more likely in crowded venues or within a household, our findings combined with others suggest that beverages, if contaminated with SARS-CoV-2 during handling, serving, or through sharing of drinks, should be considered as a potential vehicle for virus transmission.

## Figures and Tables

**Table 1:** Beverage characteristics (per 100 mL).

Beverage	Sugar (g)	Fat (g)	Protein (g)	Alcohol (%)	pH	Caffeine (mg)
Whole Milk	5.00	3.33	3.33	0.0%	6.63 ± 0.03	0.00
Skim milk	5.00	0.00	3.33	0.0%	5.42 ± 0.21	0.00
Chocolate milk	9.17	0.83	3.33	0.0%	6.96 ± 0.09	2.08
Almond milk	0.00	1.04	0.42	0.0%	8.57 ± 0.15	0.00
Coconut milk	0.04	1.88	0.00	0.0%	8.09 ± 0.15	0.00
Apple juice	11.67	0.00	0.00	0.0%	3.96 ± 0.06	0.00
White grape juice	15.00	0.00	0.00	0.0%	3.42 ± 0.11	0.00
Red grape juice	14.92	0.00	0.00	0.0%	3.57 ± 0.05	0.00
Cranberry juice	9.80	0.00	0.00	0.0%	3.10 ± 0.36	0.00
Smoothie	11.55	0.00	0.00	0.0%	3.66 ± 0.18	0.00
Pedialyte	2.50	0.00	0.00	0.0%	4.14 ± 0.00	0.00
Coffee	0.00	0.00	0.00	0.0%	5.35 ± 0.14	56.96
Tea	0.00	0.00	0.00	0.0%	5.30 ± 0.02	18.99
Dark soda	11.26	0.00	0.00	0.0%	2.61 ± 0.22	9.57
Light soda	10.81	0.00	0.00	0.0%	3.23 ± 0.17	0.00
Energy drink	10.70	0.00	0.00	0.0%	3.37 ± 0.11	31.27
Club soda	0.00	0.00	0.00	0.0%	5.30 ± 0.31	0.00
Water	0.00	0.00	0.00	0.0%	5.95 ± 0.51	0.00
Porter	0.28	0.00	0.56	6.9%	4.57 ± 0.05	0.00
Stout	1.41	0.00	0.56	6.0%	4.78 ± 0.14	0.00
IPA	0.11	0.00	0.28	6.9%	4.33 ± 0.02	0.00
Lager	2.54	0.00	0.00	5.2%	4.51 ± 0.17	0.00
Cheap light beer	0.08	0.00	0.20	4.2%	4.01 ± 0.14	0.00
Non-alcoholic beer	0.00	0.00	0.56	5.0%	4.45 ± 0.06	0.00
Hard cider	5.07	0.00	0.00	5.0%	3.37 ± 0.04	0.00
Sweet white wine	7.70	0.00	0.07	10.0%	3.09 ± 0.09	0.00
Dry white wine	0.00	0.00	0.07	12.0%	3.20 ± 0.14	0.00
Sweet red wine	4.05	0.00	0.07	14.7%	3.40 ± 0.01	0.00
Dry red wine	0.61	0.00	0.07	13.0%	3.36 ± 0.06	0.00
Whiskey	0.00	0.00	0.00	40.0%	3.91 ± 0.01	0.00
Rum	0.00	0.00	0.00	40.0%	4.36 ± 0.17	0.00
Tequila	0.00	0.00	0.00	40.0%	4.32 ± 0.25	0.00
Gin	0.00	0.00	0.00	40.0%	5.55 ± 0.31	0.00
Vodka	0.00	0.00	0.00	40.0%	5.38 ± 0.20	0.00
Cocoa powder (10%)	0.00	1.30	0.00	0.0%	5.39 ± 0.12	22.22
Cocoa powder (5%)	0.00	0.65	0.00	0.0%	5.43 ± 0.07	11.10
Cocoa powder (2.5%)	0.00	0.33	0.00	0.0%	5.62 ± 0.09	5.56
Vodka + soda (alcohol 20%)	0.00	0.00	0.00	20.0%	6.45 ± 0.23	0.00
Vodka + soda (alcohol 10%)	0.00	0.00	0.00	10.0%	6.18 ± 0.40	0.00
Saliva (vaccinated)	0.00	0.00	0.00	0.0%	7.47 ± 0.53	0.00
Saliva (unvaccinated)	0.00	0.00	0.00	0.0%	7.16 ± 0.03	0.00
Media (DMEM)	0.45	0.00	0.16	0.0%	7.58 ± 0.03	0.00

Note: Information on sugar, fat, protein, alcohol, and caffeine for each beverage were obtained from nutritional fact labels, with the exception of pH, which was measured for each beverage (pH values ± SD of each beverage, n = 3).

## Data Availability

Data is available upon request.
